# Differential Expression of Alpha-Synuclein in Hippocampal Neurons

**DOI:** 10.1371/journal.pone.0089327

**Published:** 2014-02-25

**Authors:** Katsutoshi Taguchi, Yoshihisa Watanabe, Atsushi Tsujimura, Harutsugu Tatebe, Seiji Miyata, Takahiko Tokuda, Toshiki Mizuno, Masaki Tanaka

**Affiliations:** 1 Department of Basic Geriatrics, Kyoto Prefectural University of Medicine, Kawaramachi-Hirokoji, Kamikyo-ku, Kyoto, Japan; 2 Department of Neurology, Kyoto Prefectural University of Medicine, Kawaramachi-Hirokoji, Kamikyo-ku, Kyoto, Japan; 3 Department of Applied Biology, Kyoto Institute of Technology, Matsugasaki, Sakyo-ku, Kyoto, Japan; 4 Department of Molecular Pathobiology of Brain Diseases, Kyoto Prefectural University of Medicine, Kawaramachi-Hirokoji, Kamikyo-ku, Kyoto, Japan; University of Pittsburgh, United States of America

## Abstract

α-Synuclein is the major pathological component of synucleinopathies including Parkinson's disease and dementia with Lewy bodies. Recent studies have demonstrated that α-synuclein also plays important roles in the release of synaptic vesicles and synaptic membrane recycling in healthy neurons. However, the precise relationship between the pathogenicity and physiological functions of α-synuclein remains to be elucidated. To address this issue, we investigated the subcellular localization of α-synuclein in normal and pathological conditions using primary mouse hippocampal neuronal cultures. While some neurons expressed high levels of α-synuclein in presynaptic boutons and cell bodies, other neurons either did not or only very weakly expressed the protein. These α-synuclein-negative cells were identified as inhibitory neurons by immunostaining with specific antibodies against glutamic acid decarboxylase (GAD), parvalbumin, and somatostatin. In contrast, α-synuclein-positive synapses were colocalized with the excitatory synapse marker vesicular glutamate transporter-1. This expression profile of α-synuclein was conserved in the hippocampus *in vivo*. In addition, we found that while presynaptic α-synuclein colocalizes with synapsin, a marker of presynaptic vesicles, it is not essential for activity-dependent membrane recycling induced by high potassium treatment. Exogenous supply of preformed fibrils generated by recombinant α-synuclein was shown to promote the formation of Lewy body (LB) -like intracellular aggregates involving endogenous α-synuclein. GAD-positive neurons did not form LB-like aggregates following treatment with preformed fibrils, however, exogenous expression of human α-synuclein allowed intracellular aggregate formation in these cells. These results suggest the presence of a different mechanism for regulation of the expression of α-synuclein between excitatory and inhibitory neurons. Furthermore, α-synuclein expression levels may determine the efficiency of intracellular aggregate formation in different neuronal subtypes.

## Introduction

α-Synuclein is one of the major components of Lewy bodies (LBs) and Lewy neurites (LNs), which are pathological hallmarks of synucleinopathies including Parkinson's disease (PD) and dementia with Lewy bodies (DLB) [1]–[3]. Several mutations in the α-synuclein gene are responsible for familial PD [4]–[6]. Therefore, insights into the mechanisms underlying α-synuclein pathology are crucial for the understanding of these neurodegenerative disorders. To elucidate the mechanisms of neuronal loss in these diseases, it is also important to determine the physiological roles of α-synuclein in normal neurons.

α-Synuclein is localized at presynapses *in vivo* and *in vitro* [7]–[9]. It has been suggested that α-synuclein is involved in the generation and maintenance of synapses, because this protein appears earlier than synaptophysin during development of the central nervous system and is localized to presynaptic terminals throughout the adult mammalian brain [10], [11]. α-Synuclein directly binds to synaptobrevin-2 in presynaptic regions and functions to sustain soluble N-ethylmaleimide-sensitive factor attachment protein receptor (SNARE)-complex assembly *in vivo* and *in vitro* [12]. In addition, in a recent study using an overexpression and knockout model *in vitro*, it was reported that α-synuclein maintains the size of the vesicular recycling pool [13].

In studies of α-synuclein pathogenicity, overexpression of α-synuclein in neurons resulted in formation of inclusion bodies and neuronal loss [14], and decreased the survival and dendritic development of newborn neurons [15]. In humans, the number of α-synuclein-positive neurons in the hippocampus of DLB patients is significantly increased [15]. In addition, parvalbumin-containing neurons in the neocortex are reported to be free of LBs in DLB patients [16]. These results suggest that an increase in the intracellular amount of α-synuclein is a probable risk factor for neurodegeneration, and that the mechanisms underlying aggregate formation are distinct in different neuronal cell types.

In the present study, we investigated the precise expression level and subcellular localization of α-synuclein in hippocampal neuronal cell cultures, and verified that there is a difference in the expression of α-synuclein between excitatory and inhibitory neurons. We also examined activity-dependent synaptic vesicular recycling following treatment with high potassium. A recent report demonstrated that preformed fibrils generated by recombinant α-synuclein can promote the formation of LB-like aggregates containing endogenous α-synuclein [17]. We also treated cells with these preformed fibrils, and explored whether aggregate formation depends on the expression level of endogenous α-synuclein. Finally, we examined the expression of α-synuclein in the mouse hippocampus *in vivo* and confirmed the observations of differential expression found *in vitro*.

## Materials and Methods

### Animals

C57BL/6N mice were used, and all experimental designs and procedures were approved by the Committee for Animal Research, Kyoto Prefectural University of Medicine (M23-241), Kyoto Japan, and followed the guidelines of the Ministry of Education, Culture, Sports, Science, and Technology of Japan.

### Antibodies

For α-synuclein detection, mouse monoclonal antibody Syn-1 (BD Biosciences, San Diego, CA, USA) and rabbit polyclonal antibody C-20 (Santa Cruz Biotechnology, Dallas, TX, USA) were used. For specific detection of human α-synuclein, mouse monoclonal antibody Syn211 (Thermo Fisher Scientific, UK) was used, because Syn211 recognizes human α-synuclein, but not mouse α-synuclein [18]. Phosphorylated α-synuclein was detected by mouse monoclonal antibody pSyn#64 (Wako Pure Chemical Industries, Japan) or rabbit polyclonal antibody phospho-S129 (Abcam, UK). Glutamic acid decarboxylase (GAD) was detected by rabbit polyclonal antibodies purchased from Sigma-Aldrich (St Louis, MO, USA). Anti-synapsin antibody was produced as previously described [19]. The following antibodies were purchased from these manufacturers: anti-synaptotagmin (Developmental Studies Hybridoma Bank, Iowa city, IA, USA), anti-vesicular glutamate transporter-1 (vGluT-1; Millipore, Billerica, MA, USA), anti-parvalbumin (Sigma-Aldrich), anti-somatostatin (Millipore), and anti-NeuN (Millipore).

### Cell culture and transfection

Dissociated cells prepared from hippocampi of mouse embryos (E16-18) were disseminated onto polyethylimine coated coverslips and cultured in Neurobasal medium (Gibco, Life Technologies, Carlsbad, CA, USA) supplemented with B-27 (Gibco), L-glutamine (Nacalai Tesque, Kyoto, Japan) and penicillin/streptomycin (Nacalai Tesque). In the occasion of neuronal culture without glial cells, cells cultured for 2 days were treated with a medium containing cytosine arabinoside (AraC, 10 µM) (Sigma-Aldrich) for 12 h, followed by replacement with a complete medium. Half of the volume of medium was changed every 4 days. Unless specifically mentioned, cells were cultured without AraC treatment and most experiments were performed at 16–22 days *in vitro*.

To form intracellular aggregates containing human α-synuclein, transfection of plasmid DNA for the expression of human α-synuclein was performed using the electroporator CUY21Pro-Vitro (Nepagene, Chiba, Japan) in accordance with the manufacturer's protocol. After transfection, cells were disseminated onto coverslips. For depolarizing the neuronal cell membrane, cultured cells were treated with fresh medium containing 100 mM potassium chloride for 30 min. After depolarization, cells were treated with original complete medium for 10 min, to analyze the recovery of the cell membrane.

### Immunocytochemistry

Cells were fixed with 2% paraformaldehyde (PFA) in culture medium for 10 min at room temperature. The fixed cells were permeabilized with 0.1% Triton X-100 in phosphate-buffered saline (PBS, pH 7.4) for 10 min, and blocked with 5% normal goat serum (NGS) in PBS for 30 min. Cells were then incubated with primary antibody in the blocking solution for 1–2 h. Cells were then washed with PBS and treated with a secondary antibody. For double staining, this staining procedure was repeated. The primary antibodies were detected with Alexa488- and Alexa594-conjugated secondary antibodies (Molecular Probes, Life Technologies, Carlsbad, CA, USA). For detection of the primary antibody produced in guinea pig, a fluorescein isothiocyanate-conjugated secondary antibody (Vector Laboratories, Burlingame, CA, USA) was used. Cells were washed with PBS and then with milliQ water (Millipore), and mounted with FluorSave (Millipore). Images were acquired as Z stacks (20–30 z-sections, 0.3–0.5 µm apart, 1024×1024 pixels) using a Plan-Apochromat 63x/1.40 Oil DIC objective (Carl Zeiss, Oberkochen, Germany) with an inverted laser-scanning confocal microscope, LSM510 (Carl Zeiss).

### Formation of intracellular aggregates of α-synuclein

Neurons containing intracellular aggregates of α-synuclein were obtained using previously described methods [17]. Briefly, following sonication, preformed fibrils prepared from human or mouse α-synuclein were suspended in culture medium, and the final concentration was adjusted to 5 µg/ml. Cultured cells were treated with complete medium containing the fibrils for 7 days.

### Fibril preparation

For preparation of recombinant human and mouse α-synuclein from bacterial culture, plasmid vectors were constructed as described previously (Tatebe et al., 2010; Watanabe et al., 2012). Briefly, PCR fragments of human and mouse α-synuclein were inserted into pTrc-His-TOPO vector (Invitrogen, Life Technologies, Carlsbad, CA, USA). Recombinant α-synuclein with a His-tag was purified by His-Accept column (Nacalai Tesque) [20], [21]. Purified protein was electrophoresed and the protein band was stained by Coomassie Brilliant Blue and the purity confirmed by SDS-PAGE. Fibril forms of α-synuclein were prepared in accordance with a previous report [17] with slight modifications. Briefly, fibrils of α-synuclein were generated by incubating purified α-synuclein in PBS (final solution 2 mg/ml) at 37°C with constant agitation for 7 days. After agitation, the solution containing fibrils was ultracentrifuged at 200,000×g for 2 h. Fibrils were recovered as a precipitate at the bottom of the centrifuge tube. The resulting pellet was dissolved in the original volume of PBS and stored at −20°C until use. The supernatant was also stored. Each sample was then subjected to western blot analysis.

### Construction of plasmid vector

For expression of human α-synuclein in cultured neurons, α-synuclein was amplified from a human cDNA library with the primers 5′-CGCCACCATGGATGTATTCATGAAA-3′ and 5′-GATATCTTAGGCTTCAGGTTCGTAG-3′. The PCR fragment was digested with *NotI* and then inserted into the *NotI*-*EcoRV* site of the pcDNA3.1/myc-HisB vector. The vector was then sequenced for confirmation.

### SDS-PAGE and western blotting

Samples were denatured by heating with SDS-sample buffer (62.5 mM Tris-HCl, pH 6.8, 2% sodium dodecyl sulfate, 0.003% Pyronin Y, and 10% glycerol) containing 1% 2-mercaptoethanol at 98°C for 3 min. Proteins were separated by 15% polyacrylamide gel, and transferred to polyvinylidenedifluoride membranes (Bio-Rad Laboratories, Hercules, CA, USA). Membranes were blocked with 5% skim milk in Tris-buffered saline (TBS, pH 7.4) containing 0.1% Tween-20 for 1 h at room temperature. After treatment with primary antibody, the membrane was incubated with alkaline phosphatase-conjugated secondary antibodies. Protein bands were detected using the NBT-BCIP system (NacalaiTesque).

### Image processing

The digital images obtained on the LSM510 were processed using Adobe Photoshop 7.0. Projections of Z stack images were processed with LSM Image Browser (Carl Zeiss).

### Quantitative analysis

For quantification of the ratio of α-synuclein-expressing cells or aggregate-containing GAD-positive cells, two independent cell cultures were performed and three coverslips per culture were subjected to the immunocytochemical procedures. Endogenous α-synuclein and its aggregates were detected by Syn-1 and pSyn#64 antibodies, respectively. GAD-positive cell bodies (more than 75 cells per coverslip) were subjected to the cell counting procedure. For quantification of the ratio of GAD-positive cells having intracellular aggregates composed of transfected human α-synuclein, two coverslips per culture were subjected to the immunocytochemical procedures. Human α-synuclein was detected by the Syn211 antibody, followed by counting the number of GAD-positive neurons harboring human α-synuclein aggregates. These experiments were independently repeated twice. Data represent mean ± SEM. Statistical significance of the difference in the ratio of intracellular aggregate formation was analyzed using Graphpad Prism 5 with an unpaired, two-tailed Student's t test with or without Welch's correction.

After acquisition of the confocal images, quantitative colocalization analysis using Pearson's correlation test was performed (LSM Image Browser, Carl Zeiss).

### Immunohistochemistry

Under deep anesthesia with pentobarbital, adult mice were intracardially perfused with PBS followed by 4% PFA in PBS. Brains were dissected into blocks including the cerebral cortex and hippocampus, and post-fixed with the same fixative for 12 h at 4°C. Coronal sections (40 µm) were obtained using a vibratome (DSK, Kyoto, Japan). Sections were permeabilized with 0.3% Triton X-100 in PBS (PBST) for 1 h, and blocked with 10% NGS in PBST for 6 h. Sections were then treated with primary and secondary antibodies following the immunocytochemical procedures described above. After these treatments, the sections were washed with PBST and then with 20 mM Tris-HCl buffer, and mounted with FluorSave. Images were acquired as Z stacks (10–20 z-sections, 1 µm apart, 1024×1024 pixels) using a Plan-Apochromat 63x/1.40 Oil DIC objective with an inverted laser-scanning confocal microscope, LSM510.

## Results

### Inhibitory neurons showed low expression of α-synuclein *in vitro*

**Figure 1:**
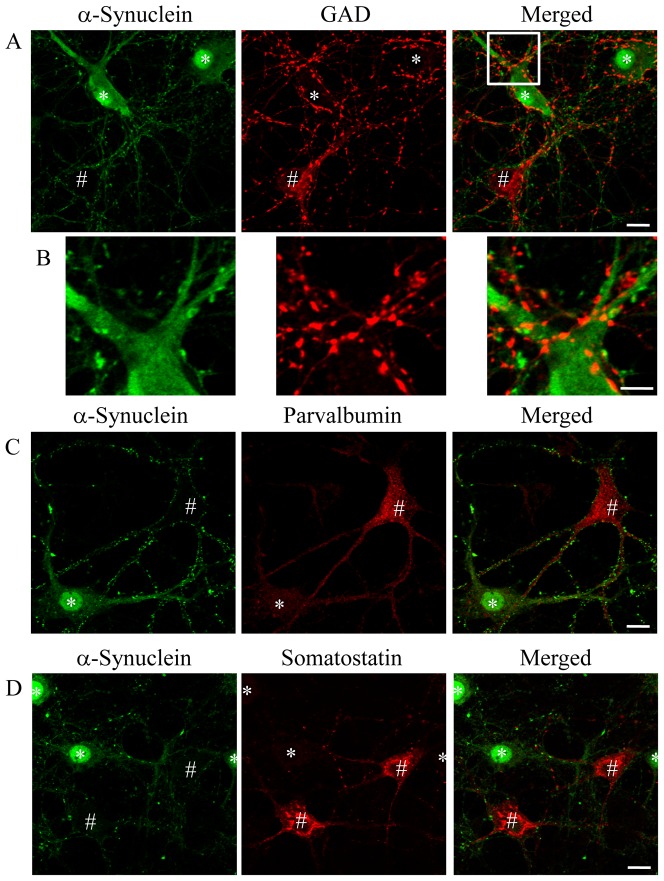
Low expression of α-synuclein in inhibitory neurons. Cells expressing the inhibitory neuronal marker proteins GAD (A), parvalbumin (C), and somatostatin (D) (indicated by #) showed low expression of α-synuclein. Cells with high expression of α-synuclein are labeled with asterisks. The region marked by a white square in A is magnified in B. Immunoreactivity of GAD was not colocalized with that of α-synuclein (B). Three independent cultures were performed and the differential expression pattern of α-synuclein was reproducibly confirmed. These images were further subjected to the quantitative colocalization analyses shown in Fig. S2. Scale bars: 10 µm in A, C, and D; 5 µm in B.

Immunocytochemistry of cultured hippocampal neurons showed that α-synuclein was present in a punctate distribution, with an intense signal observed in the cell body and nucleus of some neurons (Fig. 1A). However, other neurons were unstained with either monoclonal or polyclonal antibodies against α-synuclein (Fig. 1 and Fig. S1A). There were clearly differential expression patterns of α-synuclein in cultured neurons. To characterize the neurons with low expression of α-synuclein, we performed immunostaining using a specific antibody against GAD, a well-known marker of inhibitory neurons. GAD-positive neurons were immunonegative for α-synuclein (Fig. 1A). Although GAD-positive puncta were juxtaposed with the α-synuclein-positive dendrites, most GAD signals were not colocalized with α-synuclein (Fig. 1A, merged images). 89±1.4% of GAD-positive neurons displayed no expression of α-synuclein (6 coverslips were analyzed). About 10% of the GAD-positive neurons, however, showed faint immunoreactivity for α-synuclein. Double immunofluorescence with antibodies against α-synuclein and parvalbumin or somatostatin, other marker proteins of inhibitory neurons, confirmed that α-synuclein was not detected in parvalbumin- or somatostatin-positive neurons (Fig. 1C and 1D). These results indicated that inhibitory neurons show very low expression of α-synuclein *in vitro*. Even after depletion of glial cells by AraC treatment, this differential expression pattern of α-synuclein was still observed (data not shown).

### Cultured neurons exhibited differential expression of α-synuclein within 30 h after cell dissemination

**Figure 2:**
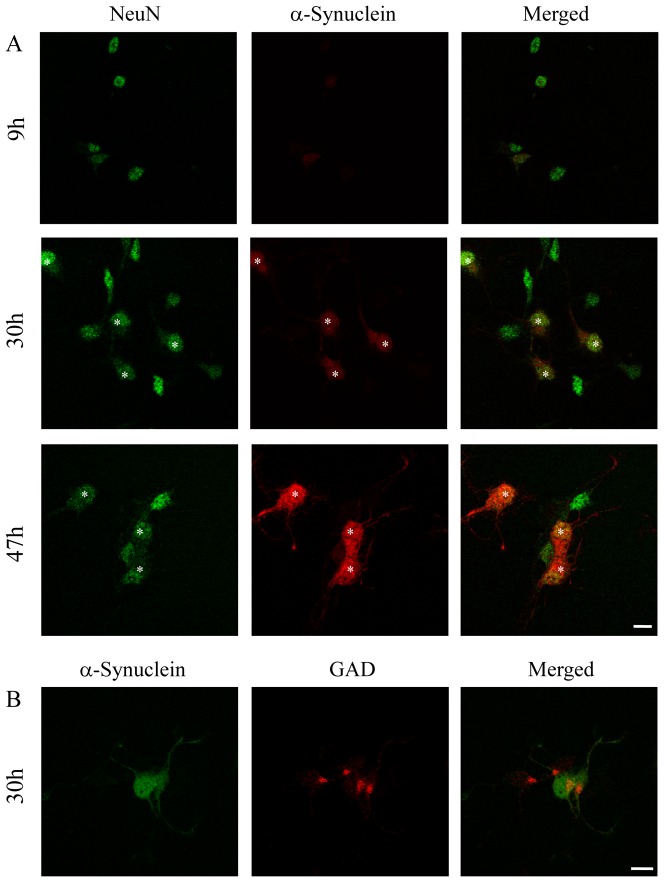
Differential expression of α-synuclein during cell maturation. (A) Expression of α-synuclein was examined at the indicated times after cell dissemination. Immunoreactivity of α-synuclein increased during cell maturation. By 30 h after cell dissemination, some NeuN-positive cells expressed α-synuclein (asterisks), but other NeuN-positive cells did not. (B) Expression of GAD at 30 h was not sufficient to distinguish inhibitory neurons from other types of neurons. Three independent cultures were performed. Scale bars: 10 µm.

To investigate the expression of α-synuclein during cell maturation, double immunostaining for NeuN, a neuron-specific marker, and α-synuclein was performed at various times after cell-disemination (Fig. 2A). Expression of α-synuclein gradually increased during cell maturation, and after 30 h cultured neurons already exhibited differential expression levels of α-synuclein. There were α-synuclein-negative neurons among the NeuN-positive cells. At this time point, GAD expression was not sufficient to distinguish inhibitory neurons (Fig. 2B).

### α-Synuclein was localized at excitatory synapses

**Figure 3:**
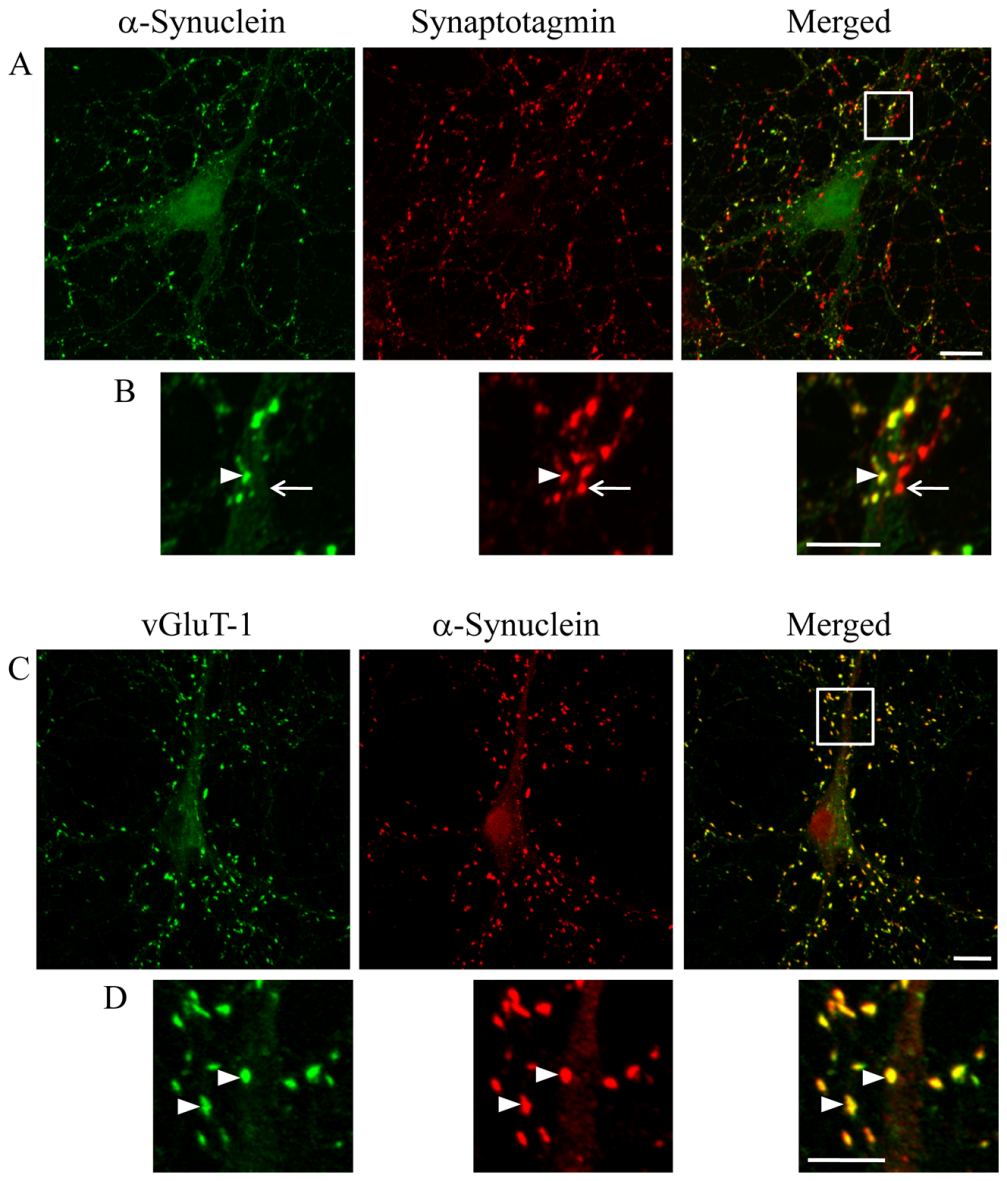
Presynaptic localization of α-synuclein in excitatory neurons. (A, B) Confocal images of double immunostaining for α-synuclein and synaptotagmin. The region marked by a white square in A is magnified in B. Arrowhead in B indicates the presynapse, expressing both α-synuclein and synaptotagmin. However, there are some synaptotagmin-positive synapses lacking α-synuclein (arrow). (C, D) Confocal images of double immunostaining for α-synuclein and vGluT-1, an excitatory neuronal marker protein. The region marked by a white square in C is magnified in D. α-Synuclein is colocalized with vGluT-1 in D (arrowheads). Three independent cultures were performed and colocalization between α-synuclein and vGluT-1 was reproducibly confirmed. Panel C was subjected to the quantitative colocalization analysis shown in Fig. S2. Scale bars: 10 µm in A and C; 5 µm in B and D.

We examined the synaptic localization of α-synuclein by double immunostaining for α-synuclein and synaptotagmin, a presynaptic marker (Fig. 3A and 3B). Although α-synuclein was colocalized with synaptotagmin in some presynapses, a subset of the synaptotagmin-positive synapses lacked α-synuclein expression. In contrast, GAD immunoreactivity was colocalized with some of the signals of synapsin, another presynaptic marker (Fig. S1B). Because α-synuclein was not co-expressed with either GAD, parvalbumin, or somatostatin in nerve terminals (Fig. 1), α-synuclein-positive puncta are not likely to be presynapses of inhibitory neurons.

Further characterization by immunostaining for vGluT-1, an excitatory synaptic marker, revealed that almost all α-synuclein-positive synapses co-expressed vGluT-1 (Fig. 3C and 3D). We also confirmed that vGluT-1 was not colocalized with GAD (Fig. S1C). Furthermore, we performed quantitative colocalization analysis using Pearson's correlation test. The correlation of pixel intensities between α-synuclein and vGluT-1 was excellent (Fig. S2A; Pearson's correlation coefficient, R = 0.83). However, the correlations of pixel intensities between α-synuclein and inhibitory neuronal markers were weak (Fig. S2B, GAD R = 0.20; Fig. S2C, parvalbumin R = 0.38; Fig. S2D, somatostatin R = 0.14). These results indicated that *in vitro* α-synuclein was predominantly expressed in excitatory neurons, but not in inhibitory neurons.

### α-Synuclein was colocalized with synapsin during presynaptic membrane recycling

**Figure 4:**
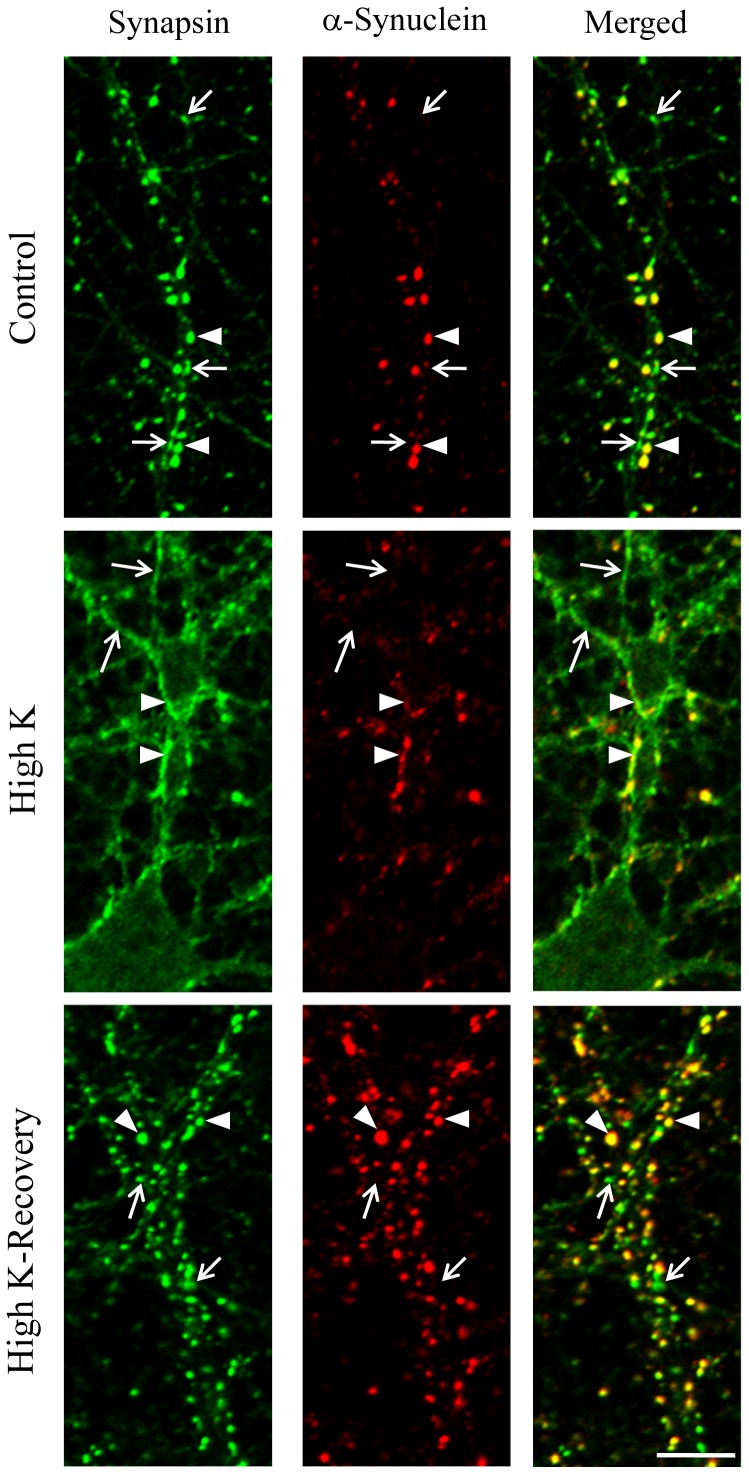
Colocalization of synapsin and α-synuclein during presynaptic membrane recycling. Synapsin immunoreactivity was observed as punctate signals before treatment (Control). After membrane depolarization by applying a medium containing high potassium, the punctate signals of synapsin changed shape and fused to the presynaptic plasma membrane (High K). The change in localization was reversed by treatment with a low-potassium medium (High K-Recovery). α-Synuclein was colocalized with synapsin during presynaptic membrane recycling (arrowheads). However, there are also instances of synapsin immunoreactivity without α-synuclein expression (arrows). Three independent cultures were performed, and these staining patterns were observed in all cultures. Scale bar: 5 µm.

Previous reports have demonstrated that α-synuclein is involved in SNARE-complex assembly and the resulting release of synaptic vesicles [12]. However, in this study some neurons were immunonegative for α-synuclein (Fig. 1). Depolarization by treatment with culture medium containing high potassium altered the synaptic localization of synapsin from synaptic vesicles to the presynaptic plasma membrane during exocytosis (Fig. 4). α-Synuclein was colocalized with synapsin during the presynaptic membrane recycling. This change in synapsin localization at synapses during depolarization also occurred in the absence of α-synuclein. This differential expression pattern was still evident at synapses following recovery from the membrane depolarization (Fig. 4). Activity-dependent recycling of synaptic membrane therefore seems to occur independently of the existence of α-synuclein.

### GAD-positive neurons were free of intracellular aggregates of α-synuclein

**Figure 5:**
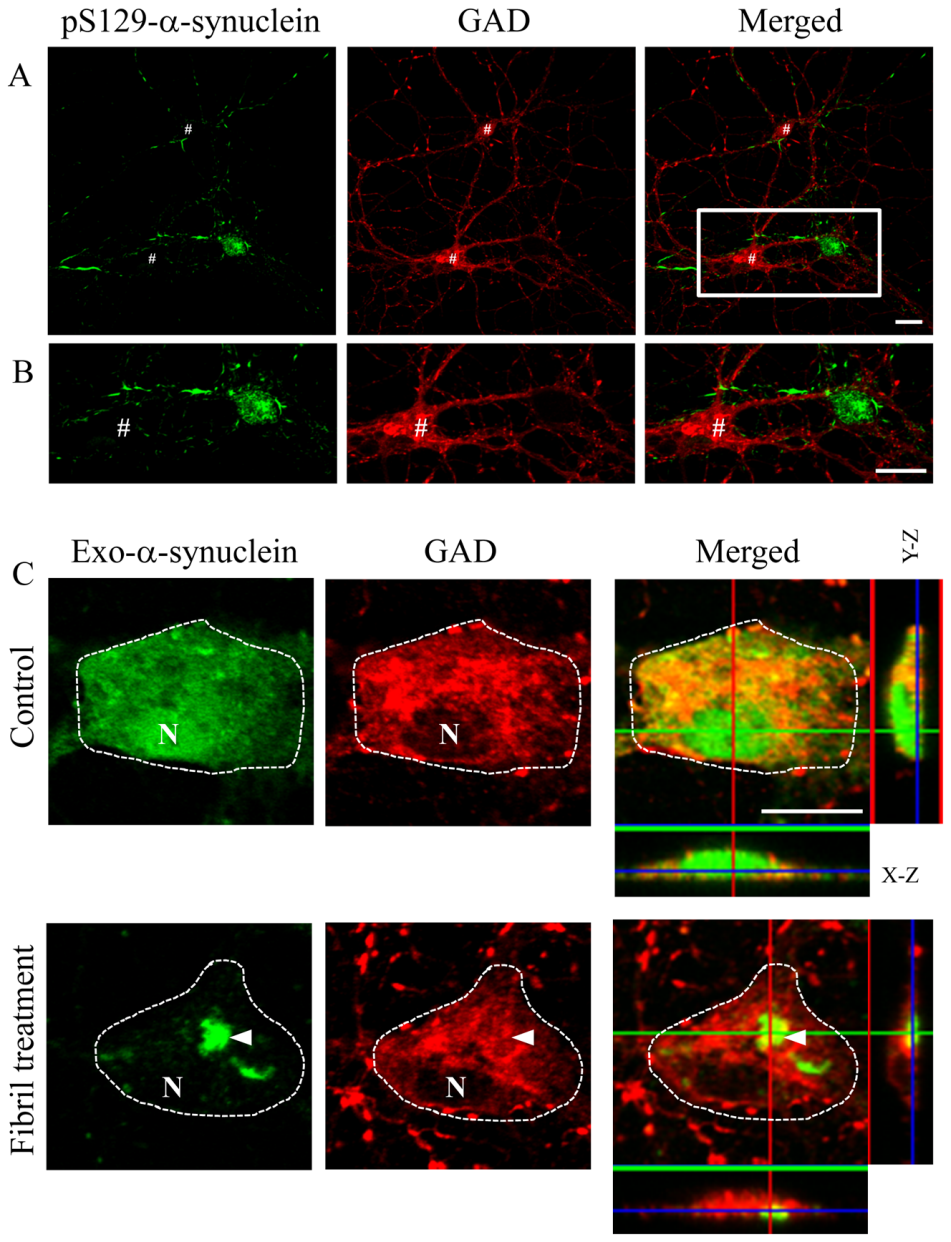
Formation of intracellular aggregates of α-synuclein. (A) Confocal images of double immunostaining for phosphorylated α-synuclein and GAD after treatment with preformed fibrils of α-synuclein. The region marked by a white square in A is magnified in B. Immunoreactivity of phosphorylated α-synuclein was observed as intracellular fibrous aggregates or inclusion bodies. GAD-positive neurons indicated by # were free of α-synuclein aggregate formation. GAD signals, including GAD-positive neurites, were not colocalized with phosphorylated α-synuclein. (C) In the absence of fibril treatment, exogenous human α-synuclein was diffusely distributed in the cell body of GAD neurons (Control). After fibril treatment, intracellular inclusions positive for α-synuclein were induced in the GAD-positive cells expressing exogenous α-synuclein. Cell bodies are shown surrounded by white dotted lines. ‘N’ indicates the location of the nucleus. Three independent cultures were performed and in all cases confirmed that intracellular inclusions were predominantly formed in GAD-negative neurons. Exogenous expression of human α-synuclein enhanced the aggregate formation in GAD-positive cells. These results were quantified and are described in Table 1. Scale bars: 10 µm.

Intracellular aggregates such as LBs and LNs are mainly composed of α-synuclein. These aggregates are formed by recruitment of intrinsic soluble α-synuclein into the insoluble aggregate core. Endogenous expression of α-synuclein, therefore, should be required for the formation of intracellular aggregates of α-synuclein [17]. Preformed fibrils prepared from recombinant α-synuclein can induce the formation of LB-like intracellular aggregates [17]. LB-like aggregates contain α-synuclein with a phosphorylated serine 129 residue [22]. If inhibitory neurons have low expression levels of α-synuclein, they may not be able to form intracellular aggregates, because of an inability to recruit endogenous α-synuclein. After treatment with preformed fibrils prepared from recombinant α-synuclein (Fig. S3), intracellular fibrous aggregates or inclusion bodies were detected by a specific antibody against phosphorylated α-synuclein (Fig.5A and 5B). This antibody can exclusively detect intracellular aggregates of α-synuclein [22]. As expected, most GAD-positive neurons were free of intracellular aggregates of α-synuclein (Fig. 5A). We also performed quantitative colocalization analysis using Pearson's correlation test. The correlation of pixel intensities between phosphorylated α-synuclein and GAD was weak (Fig. S2E; R = 0.07). The proportion of aggregate-containing GAD-positive cells was significantly lower than that of NeuN-positive cells (see note “a” in Table 1). This result is probably due to the lower amount of endogenous α-synuclein expressed in the GAD-positive cells. Another possibility is that the cell entry efficiency of the seeds derived from the preformed fibrils was different between inhibitory neurons and other types of neurons. Next, we confirmed the dose-dependent effect of the intracellular amount of α-synuclein on aggregate formation. After transfection of human α-synuclein and further treatment with preformed fibrils, intracellular accumulation of exogenous α-synuclein was observed in GAD-positive neurons. We used the Syn211 monoclonal antibody to detect only exogenous expression of human α-synuclein without detecting endogenous mouse α-synuclein, and prepared preformed fibrils from mouse α-synuclein. Before fibril treatment, exogenous human α-synuclein was diffusely expressed in the cell body (Fig. 5C). After fibril treatment, accumulation of exogenous α-synuclein was observed in some of the transfected GAD-positive cells (Fig. 5C and Table 1). The efficiency of aggregate formation was significantly increased by exogenous expression of α-synuclein (see note “b” in Table 1). These results indicated that inhibitory neurons can form intracellular aggregates under conditions where there is sufficient intracellular α-synuclein.

**Table 1:** 

Ratio	%
pS129-α-Synuclein-positive cells/NeuN-positive cells	35±0.89^a^
pS129-α-Synuclein-positive cells/GAD-positive cells	2.7±0.64^a*^
Aggregate-positive cells/GAD- and syn211-positive cells	24±2.2^b*^

a Data represent mean ± SEM (6 coverslips). Two independent cultures were performed and three coverslips per culture were used for the immunocytochemical study. Statistical analysis showed significant difference with *p*<0.0001 by student's *t* test unpaired, two-tailed.

b Data represent mean ± SEM (4 coverslips). Two independent cultures were performed and two coverslips per culture were used for the immunocytochemical study. Asterisk indicates significant difference with *p*<0.01 by student's *t* test unpaired, two-tailed with Welch's correction.

### Low expression of α-synuclein in GAD-positive cells was also conserved *in vivo*

**Figure 6:**
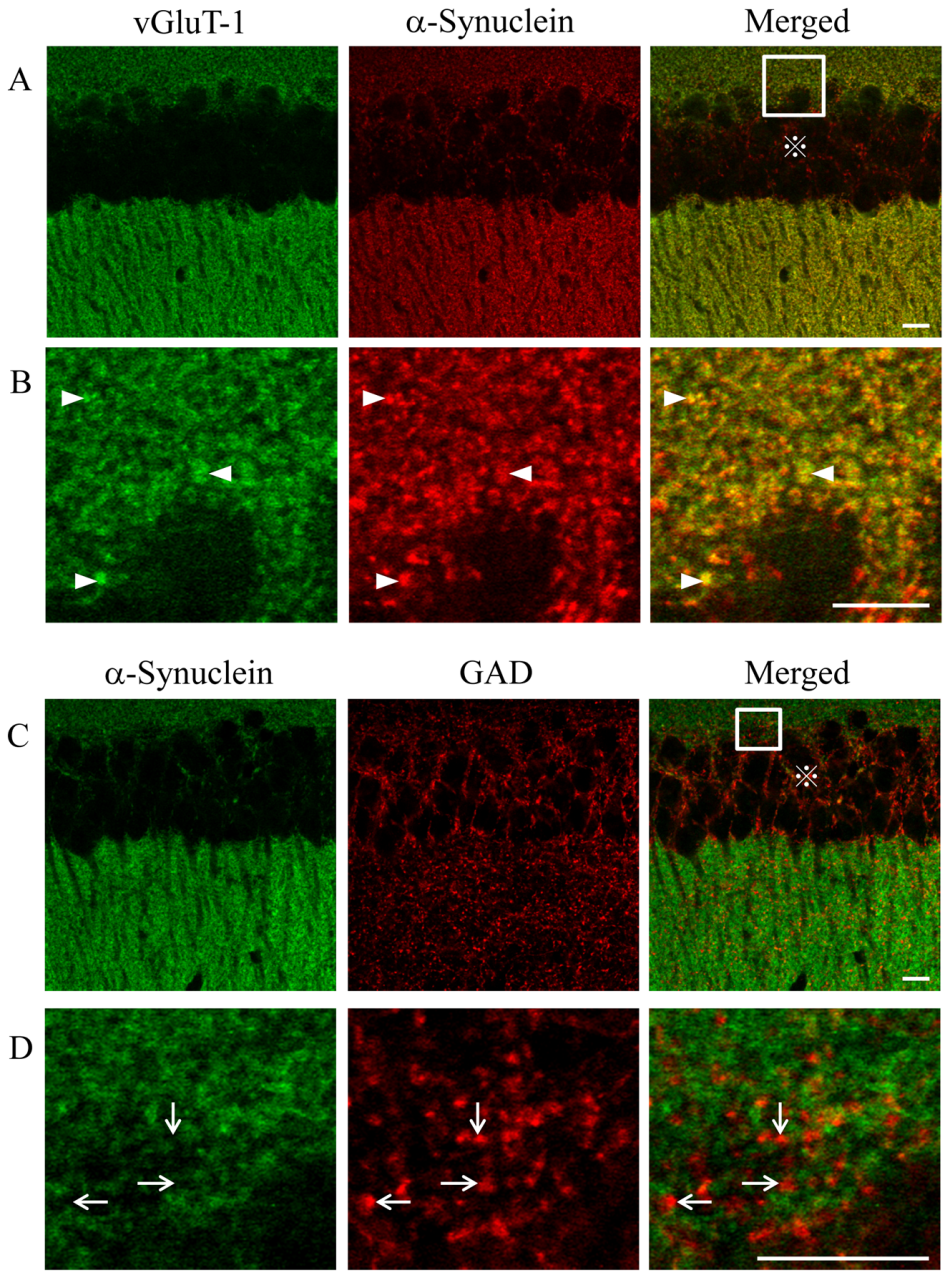
Localization of α-synuclein in the hippocampal CA1 region. (A, B) Confocal images of hippocampal neurons double immunostained for vGluT-1 and α-synuclein. The region marked by a white square in A is magnified in B. α-Synuclein is colocalized with vGluT-1 in B (arrowheads). (C, D) Confocal images double immunostained for α-synuclein and GAD. The region marked by a white square in C is magnified in D. As indicated by arrows in D, colocalization of α-synuclein and GAD was not detected. Mouse brains from two littermates were used for the immunohistochemical study. This experiment was repeated three times, and the differential expression pattern of α-synuclein was reproducibly confirmed. Panels A and C were used for the quantitative colocalization analyses shown in Fig. S2. Scale bars: 10 µm. 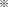 indicates stratum pyramidale.

In sections of mouse hippocampus, α-synuclein was detected as puncta in the periphery of neurons. Diffuse expression of α-synuclein was not observed in the cell body or nucleus. α-Synuclein was colocalized with vGluT-1 in the CA1 region (Fig. 6A and 6B). In contrast, α-synuclein was juxtaposed with the punctuate immunoreactivity of GAD and never colocalized with GAD signals (Fig. 6C and 6D). Furthermore, acquired confocal images were subjected to the quantitative colocalization analysis. The correlation of pixel intensities between the α-synuclein and vGluT-1 shown in Fig. 6A was excellent (Fig. S2F; R = 0.78). In contrast, the correlation of α-synuclein and GAD was weak (Fig. S2G; R = 0.11). Thus the expression pattern of α-synuclein observed in cultured inhibitory neurons was also conserved *in vivo*.

## Discussion

α-Synuclein is localized at presynapses [7], [8] and it was shown to regulate the size of the presynaptic vesicular pool in a study where antisense oligonucleotides were introduced in cultured hippocampal neurons [23]. More recently, using knockout and overexpressing neurons, Scott and Roy showed that α-synuclein plays a role in maintaining the overall size of the recycling pool of vesicles [13]. We observed expression and synaptic localization of α-synuclein in excitatory neurons, but not in inhibitory neurons, although there were no obvious differences between the two types of neurons in expression of the synaptic markers synaptotagmin and synapsin. We also observed that activity-dependent presynaptic membrane recycling (induced by high potassium treatment) occurred independently of the presence of α-synuclein.

While there have been many studies investigating the properties of postsynaptic channels including kinetics [24]–[26] and the signaling molecules involved in synaptic transduction [27], less is known about the presynaptic differences between excitatory and inhibitory neurons. Recent work revealed that the sizes of both the recycling pool and total vesicular pool are more variable at glutamatergic synapses than gamma-aminobutyric acid (GABAergic) synapses [28]. This heterogeneity of the size of the recycling pool at glutamatergic synapses may provide a dynamic range of synaptic strength that is not present at GABAergic synapses [28], [29]. α-Synuclein might act as a modulator of the size of the recycling pool at excitatory synapses. α-Synuclein is also suggested to be involved in mobilization of glutamate from the reserve pool using electrophysiology of hippocampal slices [30].

There is a possibility that the differential expression of α-synuclein is due to a difference in protein turnover between excitatory and inhibitory neurons. However, treatment with inhibitors of proteasomes or lysosomes did not alter the immunostaining patterns of α-synuclein (data not shown), suggesting that differential synthesis rather than degradation of α-synuclein is responsible for the distinct expression patterns in neurons.

Recently, it was reported that α-synuclein promotes early neurite outgrowth in cultured primary neurons [31]. It has also been suggested that α-synuclein plays important roles in the early development of synapses [10]. The expression ratio of α-synuclein/synaptophysin is higher during early development than in adult and aged rat brain [11]. We demonstrated that cultured neurons exhibit differential expression of α-synuclein by 30 h after cell dissemination. At this stage, there were no synaptic connections established between neurons. In addition, expression of GAD was very weak and not strong enough to distinguish inhibitory neurons. These results suggest that α-synuclein is involved in the differentiation of neurons.

Concerning the pathogenicity of α-synuclein, we observed that inhibitory neurons did not exhibit aggregate formation after treatment with preformed fibrils. This result was due to the low expression level of α-synuclein, because the expression of exogenous human α-synuclein in GAD neurons enabled them to form α-synuclein aggregates. A study in DLB patients showed that parvalbumin-containing cortical neurons are free of LBs and spared from degeneration, although the basal expression level of α-synuclein in these neurons was not determined [16]. In addition, it was found that overexpression of α-synuclein in a transgenic mice model caused inclusion body formation in hippocampal neurons, suggesting that high expression of α-synuclein is important for the intracellular accumulation and formation of LBs [14]. Recently, accumulation of α-synuclein was observed at the presynaptic terminals expressing vGluT-1in SNARE protein (SNAP-25) mutant mice [32].

These reports seem to be consistent with our present results. Intracellular aggregate formation composed of α-synuclein might be related to the endogenous expression level of α-synuclein, which is cell-type specific.

In conclusion, we have demonstrated differential expression patterns of α-synuclein between excitatory and inhibitory neurons *in vitro*. Importantly, these observations were also confirmed *in vivo*. Further studies will elucidate how α-synuclein works differently in the synaptic machinery of excitatory and inhibitory neurons, including in the regulation of the membrane recycling pool. Further analysis of the regulation of intracellular expression of α-synuclein will provide new insights for understanding the pathological conditions of neurodegenerative disorders including PD and DLB.

## Supporting Information

Figure S1 **Double staining for NeuN and α-synuclein.** (A) Confocal images of cultured hippocampal neurons double immunostained for NeuN and α-synuclein. α-Synuclein was differentially expressed among the NeuN-positive cells. (B) GAD-immunoreactive puncta occupied a part of the synapsin-positive synapses. (C) Immunoreactivity of GAD was not colocalized with that of vGluT-1. Two independent cultures were performed and the reactivity of the antibodies was confirmed. Scale bars: 10 µm in A; 5 µm in B and C.
(TIF)

Figure S2 **Quantification of colocalization between α-synuclein and marker proteins.** (A–G) The degree of the colocalization shown in Figs. 3C, 1A, 1C, 1D, 5A, 6A, and 6C was determined using LSM colocalization analysis and quantified using Pearson's correlation coefficient (R).
(TIF)

Figure S3 **Preparation of fibrils of α-synuclein.** (A) Purity of the recombinant α-synuclein was confirmed by SDS-PAGE. Lane M indicates the molecular masses, given in kilodaltons. His-tagged α-synuclein was detected as a 20 kDa band (lane F). This fraction predominantly contained the induced protein. CBB: Coomassie Brilliant Blue staining. (B) Western blot analysis of α-synuclein. α-Synuclein was oligomerized by agitation and the insoluble aggregates with high molecular masses were recovered in the precipitant after ultracentrifugation (Lane P). Lane Or: original fraction before agitation; Lane S: supernatant after ultracentrifugation; IB: immunoblot.
(TIF)
