# Supplementary figures and images for: Differential Expression of Alpha-Synuclein in Hippocampal Neurons
Source: PLoS One. 2014 Feb 25;9(2):e89327. doi: 10.1371/journal.pone.0089327 (PMC3934906; doi:10.1371/journal.pone.0089327)

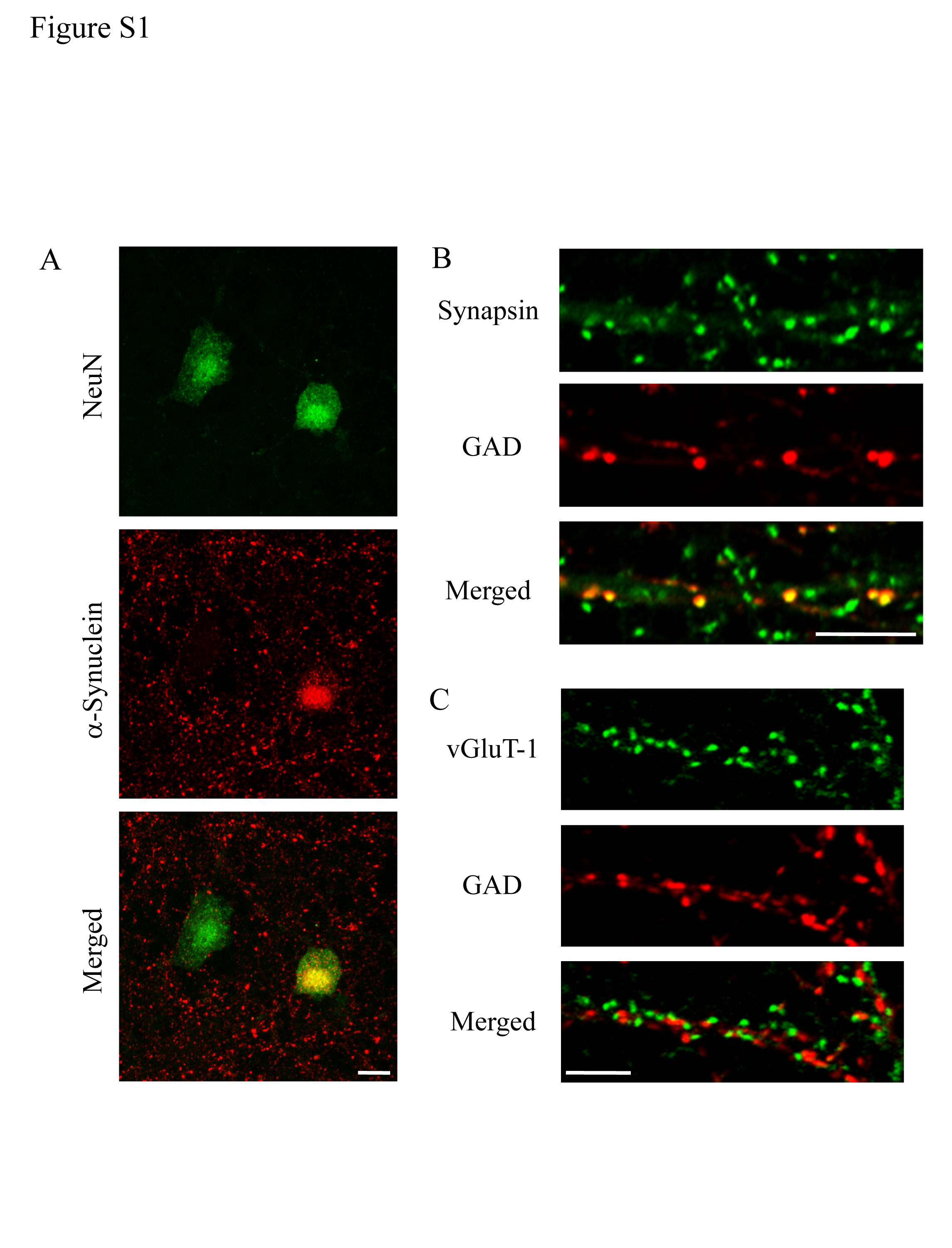

Supplement: Figure S1 — Double staining for NeuN and α-synuclein. (A) Confocal images of cultured hippocampal neurons double immunostained for NeuN and α-synuclein. α-Synuclein was differentially expressed among the NeuN-positive cells. (B) GAD-immunoreactive puncta occupied a part of the synapsin-positive synapses. (C) Immunoreactivity of GAD was not colocalized with that of vGluT-1. Two independent cultures were performed and the reactivity of the antibodies was confirmed. Scale bars: 10 µm in A; 5 µm in B and C. (TIF) [file pone.0089327.s001.tif]

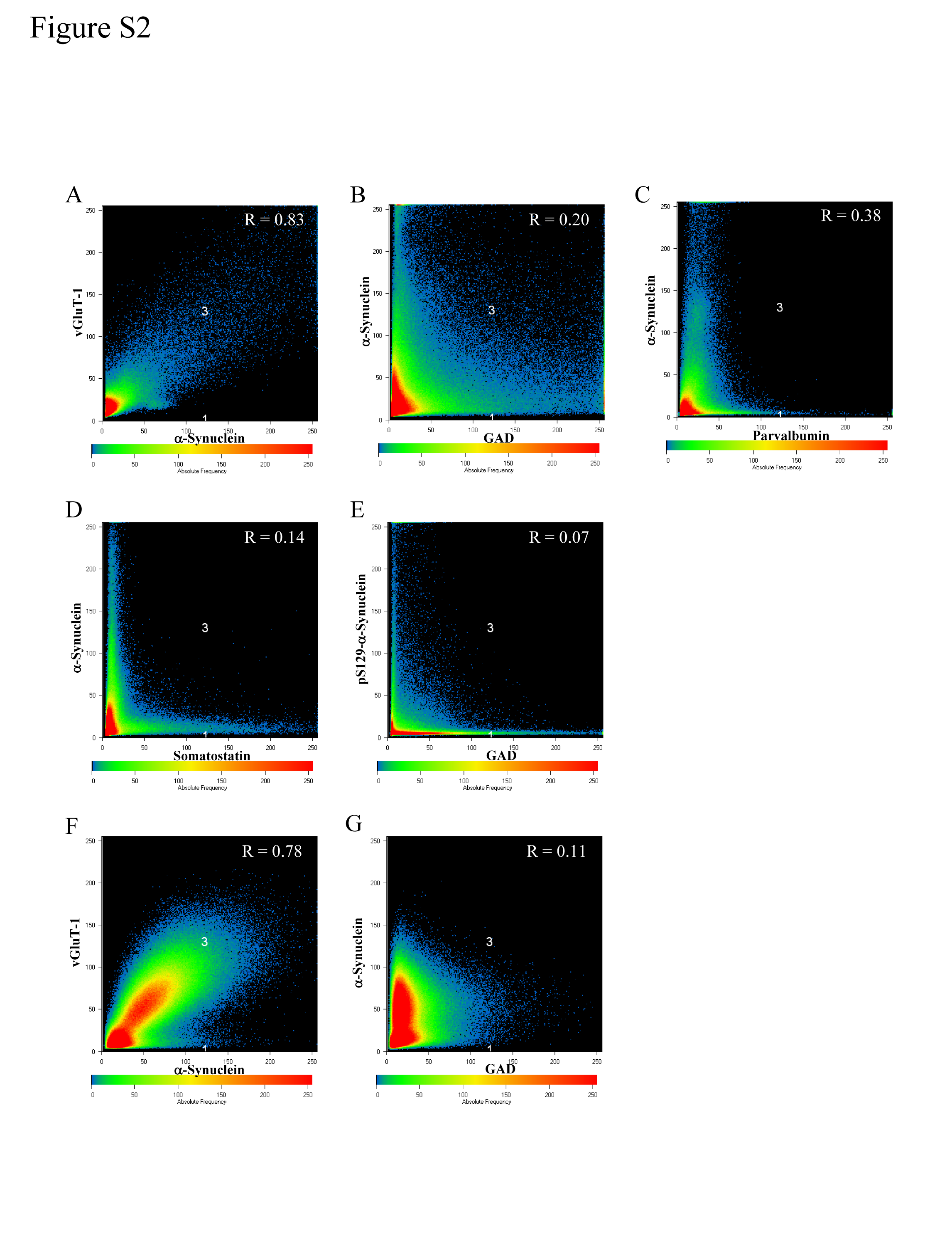

Supplement: Figure S2 — Quantification of colocalization between α-synuclein and marker proteins. (A–G) The degree of the colocalization shown in Figs. 3C, 1A, 1C, 1D, 5A, 6A, and 6C was determined using LSM colocalization analysis and quantified using Pearson's correlation coefficient (R). (TIF) [file pone.0089327.s002.tif]

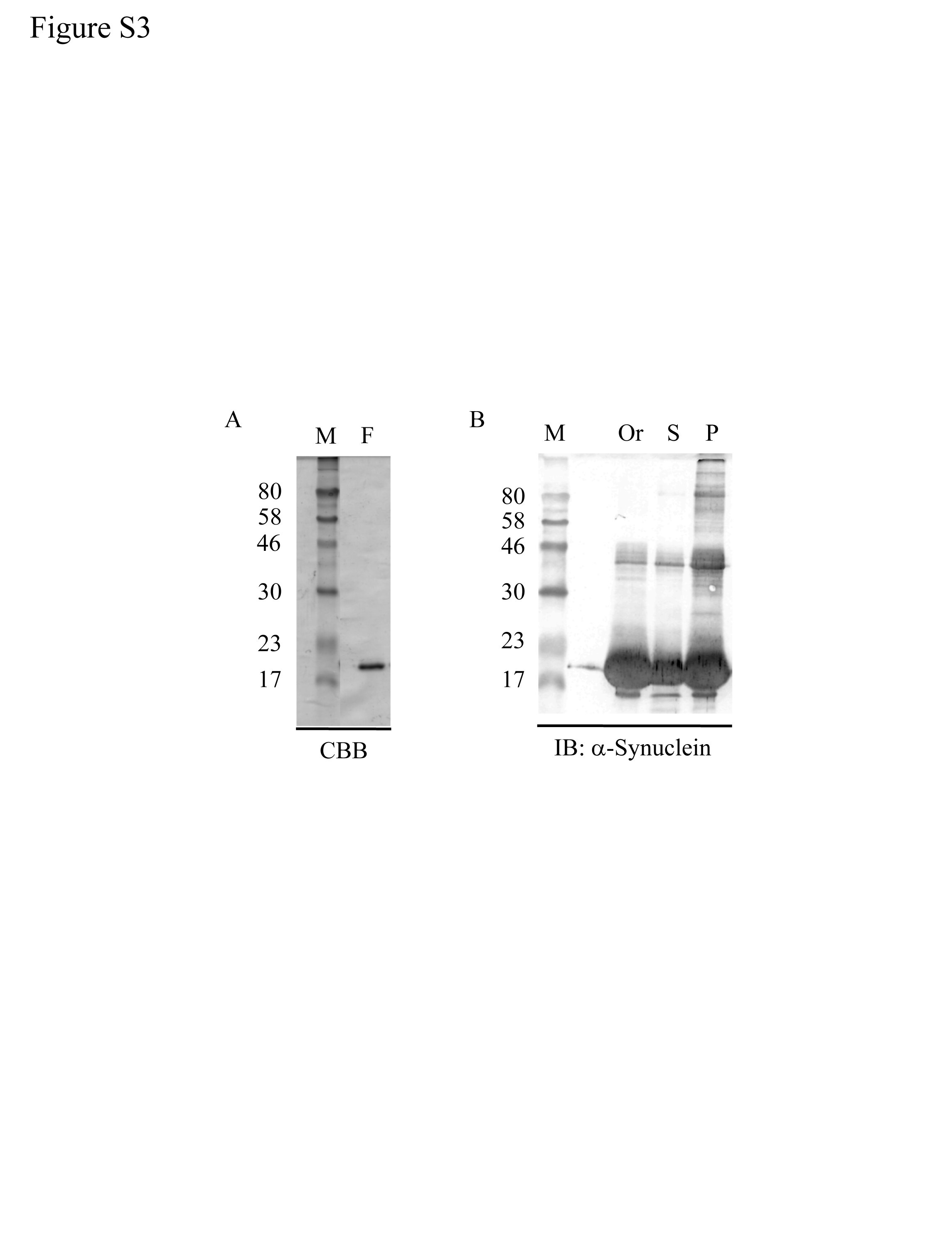

Supplement: Figure S3 — Preparation of fibrils of α-synuclein. (A) Purity of the recombinant α-synuclein was confirmed by SDS-PAGE. Lane M indicates the molecular masses, given in kilodaltons. His-tagged α-synuclein was detected as a 20 kDa band (lane F). This fraction predominantly contained the induced protein. CBB: Coomassie Brilliant Blue staining. (B) Western blot analysis of α-synuclein. α-Synuclein was oligomerized by agitation and the insoluble aggregates with high molecular masses were recovered in the precipitant after ultracentrifugation (Lane P). Lane Or: original fraction before agitation; Lane S: supernatant after ultracentrifugation; IB: immunoblot. (TIF) [file pone.0089327.s003.tif]
